# Prevalence of hepatitis B virus infection among children attending the outpatient clinic of a tertiary health centre in Southwest Nigeria

**DOI:** 10.11604/pamj.2022.43.153.35091

**Published:** 2022-11-23

**Authors:** Yetunde Toyin Olasinde, Abimbola Ololade Odeyemi, Ademola Abolarin, Efeturi Agelebe, Kehinde Joyce Olufemi-Aworinde, Joel Akande, Olufemi Idowu, Michael Alao, Olabimpe Omowumi Kofoworade, James Owolabi, Daniel Gbadero

**Affiliations:** 1 Department of Paediatrics, Bowen University, Iwo, Nigeria,; 2 Department of Haematology, Bowen University, Iwo, Nigeria,; 3 Department of Chemical Pathology, Bowen University, Iwo, Nigeria,; 4 Department of Radiology, Bowen University, Iwo, Nigeria

**Keywords:** Prevalence, hepatitis B, children, Ogbomoso, Southwest Nigeria

## Abstract

**Introduction:**

hepatitis B virus (HBV) infection is a global health disease. One-third of the world´s population is reportedly infected with the virus. Infections in children are mostly perinatal and therefore acquired early in life, with a propensity to evolve into chronic diseases and their attendant life-threatening complications. Early diagnosis can, however, improve outcomes in this group of children. The study aimed to determine the prevalence of HBV among children attending the outpatient clinic of a tertiary hospital in Southwest Nigeria.

**Methods:**

we recruited a total of one hundred and ninety-eight children aged 6 months to 18 years from the children´s outpatient clinic of a tertiary health centre, using the systematic sampling technique. HBsAg was tested using the HBsAg test kit (PRO-med®, China), and the anti-HBs antibody was tested using the ELISA method. Data were analysed using SPSS version 26.

**Results:**

of the 198 children that were screened, 2 (1.0%) were positive. Of these, one (50.0%) had a Hepatitis B positive mother and was HBeAg positive. Two-thirds of the children had received the hepatitis B vaccine, as evidenced by caregivers´ recall, or sighting of the immunization record. There was no statistically significant relationship between the hepatitis B status of the children and the sociodemographic parameters.

**Conclusion:**

the study supports the fact that paediatric HBV infections are transmitted from mother to child. Though the prevalence of HBsAg in the study population was lower than the national average for the country, routine immunization program should be strengthened for further control of HBV. Age and gender were not significantly associated with HBV infection in this study.

## Introduction

Hepatitis B virus (HBV) is a hepatotropic virus with the potential to cause life-threatening liver damage [1]. It is a disease of global health importance and is endemic in most parts of Sub-Saharan Africa; Nigeria inclusive [2]. The virus has the potential to cause chronic infections and increases the risk of death from childhood hepatic failure, cirrhosis of the liver, and liver cancer [1,2]. One-third of the World´s population is infected with HBV [1,3]. In the year 2019, the World Health Organisation (WHO) estimated that 296 million people were living with chronic HBV infection globally and also reported 820,000 deaths from it [2]. Africa as a whole is endemic to the virus and within the same country, the endemicity level varies from district to district and in different target groups [4]. Nigeria is one of the countries in the HBV hyper-endemic zone [4]. A meta-analysis of 46 Nigerian studies puts the average prevalence in the country as 13.6% [5]. The analysis also reported a prevalence of 14.0% among blood donors and 14.1% among pregnant women attending antenatal clinics [5]. In the paediatric population, differing rates have been reported from different parts of the country; 44.7% among primary school children in Borno, Northern Nigeria, 13.9% in a hospital-based study of children attending an outpatient clinic in Ilesha, South-West Nigeria, and 1.2% among adolescents attending secondary schools in Calabar, South-south Nigeria [6-8].

Childhood HBV is commonly transmitted from mother to child during the perinatal period, or through horizontal transmission via exposure to the blood of an infected individual [2,9]. The earlier HBV infection is acquired, the higher the possibility of developing a chronic infection and its sequelae. Chronic infection is more common in infants who acquire the virus perinatally from their mothers or before the age of 5 years [2,9]. To reduce the incidence of HBV infection, the WHO recommends that all infants receive the hepatitis B vaccine as soon as possible after birth, preferably within 24 hours, followed by 2 or 3 doses of hepatitis B vaccine at least 4 weeks apart [2]. The Nigerian government introduced the hepatitis B vaccine into the National Program on Immunization (NPI) schedule in the year 2004. There is limited access to diagnosis and treatment of hepatitis B in resource-poor environments like Nigeria. The WHO reported that only 10.5% of the infected people in year 2016 were aware of their HBV status [2]. Furthermore, only half of those eligible for treatment received it [10]. This implies that the majority of HBV infected people are unaware of their infection thus only a minority of treatment-eligible people have access to treatment, thereby constituting a high risk of progressing to complications such as liver cirrhosis and hepatocellular carcinoma. This study, therefore, sought to screen children attending the outpatient clinic of the Bowen University Teaching Hospital, (BUTH) Ogbomoso, to determine the prevalence of HBV infection in them and the associated social factors.

## Methods

**The study design and setting:** this was a descriptive study conducted at the Bowen University Teaching Hospital (BUTH) Ogbomoso, Oyo State, Nigeria over 5 months; between 1^st^ March 2021 and 31^st^ July 2021. The city of Ogbomoso is a pre-colonial urban center and the second-largest city, both in terms of population and geography, in Oyo State, Nigeria [11]. The institution is a Christian mission hospital that offers tertiary care to its patients. There are specialists in different departments and facilities to train medical students and residents in family medicine. The hospital also serves as a referral center to primary and secondary health care facilities in and around Ogbomoso, including neighbouring states like Osun and Kwara. The Department of Paediatrics cares for children and adolescents up to 18 years. It consists of four units, (the children´s emergency unit, the neonatal unit, the children´s outpatient unit, and the paediatric ward) from which patients are seen as either outpatients or inpatients.

**Participants:** eligible children from ages 6 months to 18 years, attending the children´s outpatient clinic of the BUTH whose caregivers gave written consent in addition to assent (in children older than 7 years) were recruited into the study. Children whose caregivers declined participation in the study were excluded.

**Sample size estimation and recruitment of study participants:** the minimum sample size required for the study was estimated using the Cochran´s formula [12]:


$$\left[n=\frac{z^{2}pq}{d^{2}}\right]$$

For this study, a prevalence of HBV infection in children (11.5%) previously reported in Nigeria [5] was used. The tolerable margin of error was set at 5% and a 10% correction for non-response was made. Thus, a minimum sample size of 173 was estimated for the study, however, 198 children eventually participated in the study. We used the systematic sampling method in the recruitment of children into the study. The sampling interval (n) was estimated by dividing our sample size by the average number of children that attended the outpatient clinic daily. Based on the average number of children, every n^th^ child on the clinic register was chosen, but the first child on each clinic day was selected by simple random method (by balloting). This was done daily till the sample size was achieved. When the parent of a child refused to give consent, the next child on the clinic register was recruited.

**Data collection instrument and method:** a semi-structured interviewer administered questionnaire was developed after reviewing similar studies and used for data collection. These were used to obtain relevant history and socio-demographic information from the parents of the subjects. The questionnaire was constructed in simple English language and was translated to Yoruba language for respondents who were more comfortable answering in their native Yoruba language. Back translation was done to the English language to preserve the original meaning of the questions. Data were collected by two research assistants, who had been trained by the principal investigator. Pretesting of the instrument was done among 20 children selected using convenience sampling method in the well-baby/immunization clinic of the BUTH, which was a different clinic from the outpatient clinic used for the main study. The pre-test exercise helped us to identify wrongly phrased items of the questionnaire and helped to assess the internal consistency of the questions. Identified ambiguous questions were either rephrased or removed entirely. The pretested questionnaires were not included in the final analysis. Informed consent was signed by the parents. In addition, assents were signed by children older than seven years. The biodata, history of any present complaint, as well as the past medical history were obtained from the parent, caregiver, or the child (if old enough to give information). History of the child´s immunization, previous blood transfusions, or history of contact with anyone with jaundice or sharing of needles was also documented. The social class of the subjects was determined using the Oyedeji classification of social class [13]. The mean of four scores (two for the father and two for the mother) to the nearest whole number was the social class assigned to the child [13]. Five milliliters (5ml) of blood sample were collected from all the selected children through an aseptic means into plain vacutainer bottles. Blood was allowed to retract, and sera were collected into labeled serum bottles after centrifugation at 3000rpm for 10 minutes and stored at -20°C till analysis.

**Laboratory analysis:** the samples were assayed for Hepatitis B Surface Antigen (HBsAg) using a commercial immunochromatographic rapid test kit (PRO-med®, China) and the markers; Hepatitis B surface antibody (anti-HBs), Hepatitis B envelope antigen (HBeAg), Hepatitis B envelope antibody (anti-HBe), and Immunoglobulin M antibody to core Hepatitis B antigen (HBcAb-IgM) were assayed with enzyme-linked immunosorbent assay (ELISA) method using a microtiter well following the manufacturer´s instructions. The kits and ELISA reagents were greater than 99.68% sensitive and 99.87% specific. The HBsAg results were reported as positive, negative, or invalid accordingly. For each invalid test, the test procedure was reviewed, and the test repeated with a new strip. The ELISA kit and its reagents were stored in the refrigerator, while the rapid test kits were stored at room temperature prior to use as directed by the manufacturers. The opened reagents that were not used immediately were refrigerated and brought to room temperature before use. Prior to analyses of the samples, ten samples were used to test run the incubator and ELISA microplate reader, adjustments were made to ensure correct results as indicated in the manufacturer's instruction. Each sample was compared with the control. Strict compliance to manufacturer's instruction for the test kits was ensured. Only clear, non-haemolyzed serum samples were analysed. Both anti-Hbs and anti-HBc antibody quantification were done to possibly detect occult HBV infection in Hbsag negative patients. Furthermore, the two positive cases were re-tested using a different commercial test kit. This was to preclude false negative and false positive results respectively.

### Data analysis

Data obtained were entered into a computer, and analysis was done using Statistical Package for Social Sciences (SPSS) version 26. Each questionnaire was field edited to ensure that the data was complete before entry into the computer. The mean and standard deviation were derived for continuous variables, and categorical variables were summarised as proportions, and further analysed using Chi-square to assess association between the variables. Chi-square was used to determine the relationship between the hepatitis B virus infection and socio-demographic variables such as age, sex, and educational status. Fisher´s exact test was used when more than 20% of expected counts were less than 5. The level of significance for all tests was set at p < 0.05 at a 95% confidence interval. Children in socioeconomic classes I&II were classified as high socioeconomic class, those in class III were classified as a middle socioeconomic class and those in classes IV and V were classified as low socioeconomic class.

### Measures and variables

**Dependent variable:** HBV status of the children.

**Independent variables:** socio-demographic characteristics (age, gender, socioeconomic status, family type, religion & tribe) history of previous blood transfusions, risky sexual behavior, scarification marks in the children.

**Ethical considerations:** approval was obtained from the Bowen University Health Research Ethics Committee (HREC) with approval number BUTH/REC-046. Written informed consent of parents or caregivers was sought and obtained before enrollment of their wards. The caregivers were appropriately educated on the significance of the research to get their optimal support.

## Results

**Figure 1:**
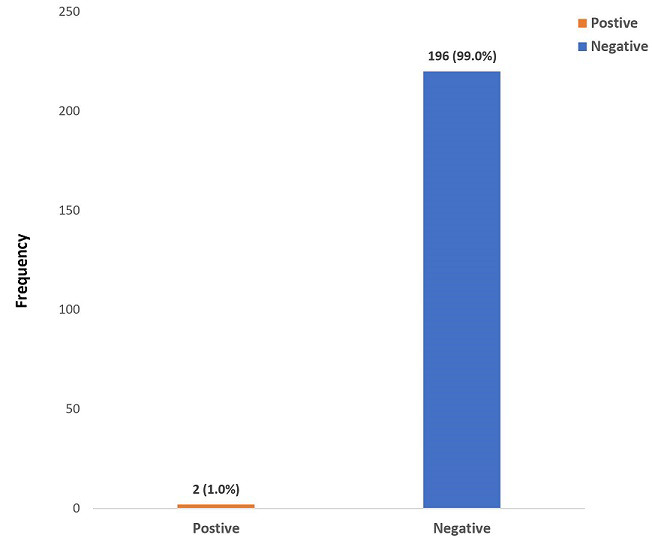
prevalence of hepatitis B

A total of 198 children were recruited into the study. The adolescents constituted the majority (68.1%) of the study population. The mean± SD age of the study population was 8.7±4.6 years. Of the children recruited into the study, 120 (60.6%) were males and 78 (39.4%) were females, with a M: F of 1: 0.7. Most 83 (41.9%) of the children were from the upper social class. The other demographic characteristics are as highlighted in Table 1. Of the screened children, 2 (1.0%) were positive for HBsAg, as shown in Figure 1. As depicted in Table 2, about two-thirds of the children had received the hepatitis B vaccine as evidenced by caregivers´ recall or sighting of the immunisation record. A third of the study population had received blood transfusion in the past. Table 3 shows the sociodemographic characteristics and laboratory parameters of the children who were positive for hepatitis B; 1 (50%) of them was vaccinated, and had a mother who was positive for hepatitis B. Furthermore, the child was positive for HBe Ag and negative for Anti-HBS antibody. The parents of the unvaccinated HbsAg positive child refused further testing. There was no statistically significant relationship between the hepatitis B status of the children and the sociodemographic parameters as shown in Table 4.

**Table 1:** sociodemographic characteristics of the study population

Variable	Frequency	Percent
**Age (years)**		
≤ 5	54	27.3
6 - 10	69	34.8
11 - 15	58	29.3
16 - 18	17	8.6
Mean ± SD	8.7 ± 4.6	
Range	1 - 18	
**Sex**		
Male	120	60.6
Female	78	39.4
**Social class**		
Upper	83	41.9
Middle	77	38.9
Lower	38	19.2

**Table 2:** risk factors for HBV infection in the study population

Variable	Frequency	Percent
**Ever received a blood transfusion**		
Yes	49	24.7
No	149	75.3
**Received Hepatitis B vaccine**		
Yes	123	62.1
No	75	37.9
**Sexually active**		
Yes	7	3.5
No	191	96.5
**Scarification/ cosmetic marks**		
Yes	17	8.6
No	181	91.4
**Engage in sharing needles or clippers**		
Yes	97	49.0
No	101	51.0

**Table 3:** sociodemographic characteristics of the positive children

Variable	Frequency	Percent
**Age (years)**		
≤ 5	1	50.0
6 - 10	1	50.0
**Sex**		
Male	1	50.0
Female	1	50.0
**Social class**		
Upper	1	50.0
Lower	1	50.0
**Know anyone with yellowness of eyes**		
Yes	1	50.0
No	1	50.0
**Ever received a blood transfusion**		
Yes	0	0.0
No	2	100.0
**Received Hepatitis B vaccine**		
Yes	1	50.0
No	1	50.0
Mothers´ hepatitis status		
Positive	1	50.0
Negative	0	0.0
Unknown	1	50.0
**Laboratory parameters**		
HBe Ag.		
Positive	1	50.0
Negative	0	0.0
Unknown	1	50.0
**Anti HBs ab**		
Positive	0	0.0
Negative	1	50.0
Unknown	1	50.0
**Anti HBc ab**		
Positive	1	50.0
Negative	0	0.0
Unknown	1	50.0
**LFT**		
Deranged	0	0.0
Not Deranged	1	50.0
Not known	1	50.0

**Table 4:** relationship between sociodemographic parameters and hepatitis status

	Hepatitis B				
	Positive	Negative	Total	χ2	p-value
Variable	n (%)	n (%)	N (%)		
**Age (years)**					
≤ 5	1 (1.9)	53 (98.1)	54	1.784F	0.795
6 - 10	1 (1.4)	68 (98.6)	69		
11 - 15	0 (0.0)	58 (100.0)	58		
16 - 18	0 (0.0)	17 (100.0)	17		
**Sex**					
Male	1 (0.8)	119 (99.2)	120	0.095F	1.000
Female	1 (1.3)	77 (98.7)	78		
**Social class**					
Upper	1 (1.2)	82 (98.8)	83	2.067F	0.498
Middle	0 (0.0)	77 (100.0)	77		
Lower	1 (2.6)	37 (97.4)	38		
**Family type**					
Monogamous	2 (1.2)	158 (98.8)	160	0.480F	1.000
Polygamous	0 (0.0)	38 (100.0)	38		
**Tribe**					
Yoruba	2 (1.1)	188 (98.9)	190	0.085F	1.000
Others	0 (0.0)	8 (100.0)	8		
**Religion**					
Christian	1 (0.7)	140 (99.3)	141	0.443F	0.494
Islam	1 (1.8)	56 (98.2)	57		

**χ2:** Chi-square test; **F:** Fisher´s exact test

## Discussion

This study found the prevalence of hepatitis B in children attending the children outpatient of the BUTH, Ogbomoso, Southwest, Nigeria to be 1.0%. This is similar to the 1.2% prevalence reported among adolescents in the south-south region of the country [8], but much less than previously reported among school children in southwest Nigeria [7]. Sadoh *et al*. [14] had also reported a much higher prevalence (13.9%) of HBV infection among sick children at the emergency unit of a tertiary centre in Benin, Southwest Nigeria. The reason for the much lower prevalence in this study might be due to the presence of two tertiary health institutions and several other primary and secondary health centres in Ogbomoso, the study centre. This could have contributed to a higher immunisation coverage among children in this city. The HBV vaccination rate in this study was much higher than previously reported among the paediatric age elsewhere in Nigeria [15]. Furthermore, it is higher than the national HBV vaccine coverage of 50% as reported by the 2018 Nigeria Demographic and Health Survey (NDHS) [16]. Accessibility and distance to vaccination facilities are recognised non-modifiable factors that influence vaccine uptake in African children [17,18]. Health education on prevention practices, early seeking of health-care assistance, and effective utilization of these health-care facilities may have played a role in this trend. Vaccination efforts among these children should however be sustained and intensified. In addition, studies conducted in urban areas of Nigeria and elsewhere showed a lower prevalence of HBV than studies conducted in rural areas of the country Ogbomoso [19,20], the study centre, is an urban area [11].

Furthermore, the introduction of the hepatitis B vaccine to the Nigerian NPI in 2004 antedates the birth of the children recruited into this study, thus the children were born in the post hepatitis B vaccine era. This could be the reason for the low prevalence of HBV infection observed in this study. Some authors (in and outside Nigeria) have reported a decline in the seroprevalence of HBV infection since the onset of the routine immunisation against hepatitis B [19,21,22]. The WHO recommends that the HBV vaccine is an effective tool in reducing the incidence of hepatitis B. This is probably responsible for the recently reported decline in the national prevalence of HBV in the country [19]. A previous study of hepatitis among pregnant women in the same region (Oyo state, southwest Nigeria) had reported that most of the pregnant women studied were Hbe antigen-negative, implying that they had a lower likelihood of transmitting the virus to their unborn babies [23]. This could also be responsible for the low HBV prevalence among children in this study.

One of the two HBV-positive children had a hepatitis B-positive mother. The child, although was vaccinated, never had hepatitis B immunoglobulin (HBIG). Mother-to-child transmission of HBV is common, a similar experience has been reported in Taiwan [22]. The WHO recommends that the infant of a hepatitis B-positive mother be given the (HBIG) and the hepatitis B vaccine within the first 12 hours of life [2]. In addition to infant vaccination, the WHO recommends the use of antiviral prophylaxis for the prevention of hepatitis B transmission from mother to child [2]. This mother was not diagnosed antenatally, hence, appropriate measures were not taken to prevent mother-to-child transmission.

The laboratory parameters in one of the HBsAg positive children (who is positive for Anti- HBc antibody) corroborates the known fact that children are mostly infected from birth and have chronic disease [2,9]. The likelihood of developing a chronic infection is more common in infants who acquire the virus perinatally from their mothers or before the age of 5 years [2,9]. This child likely acquired the infection from his HBV-positive mother. Furthermore, the normal liver enzymes in this child may portray an immune tolerant phase, in which most infected children will remain until late childhood or adolescence [24]. The positive HBeAg status in this child makes him at increased risk to transmit the disease to others and develop complications from it. Iorio *et al*. [25] reported that a quarter of the children in their cohort had not seroconverted to Anti-HBe antibody after 24 years of follow up. Previous epidemiological and clinical studies in children and adults suggest that HBeAg to anti-HBe seroconversion occurs in the third or fourth decade of life in a substantial proportion of perinatally infected HBV carriers [26,27].

Almost half of the studied children engaged in risks such as exposure to sharp objects (most of them reported sharing of hair clippers at the barbing salon). Sharing of sharps, transfusion of unsafe blood, and close contact with infected individuals are recognised risk factors for transmission of hepatitis B infection [2]. These have been previously reported as significant associated risk factors for hepatitis B infection among a cohort of children from the Southeastern part of Nigeria [15]. A small proportion of the children in our study population had scarification marks on them. Scarification marks and tattoos are common traditional practices that have both spiritual and socio-cultural values among the Yoruba tribe in the Southwestern region of Nigeria [28]. These have health implications, such as the transmission of diseases like HBV, as improperly sterilised sharps may be shared during the process of scarification or tattooing. The low prevalence of HBV infection in this study despite the risks identified among the study population, may imply that precautions (such as sterilisation of clippers at the salons, and use of new blades during the scarification process) are being taken. Emphasis should however still be intensified on information dissemination on the risks and modes of HBV transmission.

There was no observed difference in the gender risk for HBV infection, as previously reported in the same region by Sadoh *et al*. [14]. Although, Bukbuk *et al*. [6] and Isah *et al*. [29] had reported a male predominance in Northern Nigeria, and Donbraye *et al*. [7] reported a female predominance in another part of the country, they reported no statistically significant relationship between gender and hepatitis status. There appears to be no clearcut gender predisposition for hepatitis infection, thus both genders should be counselled equally on risks. Similarly, there was no observed significant relationship between hepatitis status and the age group as well as the socio-economic strata of the study population. This has been previously reported in another study [14].

## Conclusion

The prevalence of HBV infection among the children in the present study was lower than the national average for Nigeria, however, several children are still engaged in risks such as sharing of hair clippers and razor blades. There was no significant relationship between the hepatitis status and the gender, age, or socioeconomic status of the study population. We recommend strengthening of the routine immunisation, as only two-thirds of the study population in this study had received the hepatitis B vaccine. Furthermore, mass vaccination of the adults against hepatitis B will decrease the reservoir of chronic carriers and break the vicious cycle of perinatal transmission. Routine screening of pregnant women for HBV during antenatal clinics will reduce mother-to-child transmission. **Strengths of the study:** the authors ensured a robust study design to ensure that the power of the study was adequate. Furthermore, we used a scientific means to determine the sample size and recruit eligible respondents into the study. **Limitations of the study:** the information obtained from mothers could be subject to recall bias, social acceptability bias, and responder bias. We however minimised these forms of bias by maintaining anonymity with the questionnaires and by maintaining confidentiality of information given.

### What is known about this topic

- Hepatitis B infection is endemic in Nigeria;
- The hepatitis B vaccine has been introduced to tackle this problem.

### What this study adds

- This study has provided the prevalence of hepatitis B in children delivered in the post-vaccination era in Ogbomoso, Southwest Nigeria; there are no previous data on the prevalence of the HBV infection in the paediatric age group at the study site;
- This study shows that the public health intervention against HBV (the hepatitis B vaccine) needs to be further strengthened as only two-thirds of the study population had received the vaccine;
- The study also shows that several children are still engaged in risks such as sharing of hair clippers and razor blades bringing out the need for more awareness on risk factors for hepatitis; age and gender are not significantly associated with hepatitis status.
